# Graph autoencoder with mirror temporal convolutional networks for traffic anomaly detection

**DOI:** 10.1038/s41598-024-51374-3

**Published:** 2024-01-13

**Authors:** Zhiyu Ren, Xiaojie Li, Jing Peng, Ken Chen, Qushan Tan, Xi Wu, Canghong Shi

**Affiliations:** 1 The College of Computer Science Chengdu University of Information Technology, Chengdu, 610225 China; 2 Sichuan Digital Transportation Technology Co., Ltd, Chengdu, China; 3 School of Computer and Software Engineering, Xihua University, Chengdu, 610039 China

**Keywords:** Civil engineering, Information technology, Scientific data

## Abstract

Traffic time series anomaly detection has been intensively studied for years because of its potential applications in intelligent transportation. However, classical traffic anomaly detection methods often overlook the evolving dynamic associations between road network nodes, which leads to challenges in capturing the long-term temporal correlations, spatial characteristics, and abnormal node behaviors in datasets with high periodicity and trends, such as morning peak travel periods. In this paper, we propose a mirror temporal graph autoencoder (MTGAE) framework to explore anomalies and capture unseen nodes and the spatiotemporal correlation between nodes in the traffic network. Specifically, we propose the mirror temporal convolutional module to enhance feature extraction capabilities and capture hidden node-to-node features in the traffic network. Morever, we propose the graph convolutional gate recurrent unit cell (GCGRU CELL) module. This module uses Gaussian kernel functions to map data into a high-dimensional space, and enables the identification of anomalous information and potential anomalies within the complex interdependencies of the traffic network, based on prior knowledge and input data. We compared our work with several other advanced deep-learning anomaly detection models. Experimental results on the NYC dataset illustrate that our model works best compared to other models for traffic anomaly detection.

## Introduction

In recent years, intelligent transportation have become increasingly complex due to rapid urbanization and population growth. There is a growing need for abnormal event detection in transportation networks. The Shanghai Bund trampling incident that occurred on December 31, 2014, in China is a widely known tragedy closely associated with traffic anomaly detection^1^. Furthermore, on January 26, 2017, in Harbin, the largest city in northeastern China, a single traffic incident resulted in a chain of rear-end collisions, leading to eight fatalities and thirty-two injuries^2^. The above events show that early detection and prediction of anomalies before they occur are of significant value in preventing serious incidents. Therefore, an efficient and accurate anomaly detection system holds significant research value as it enables continuous monitoring of specific indicators and effective prevention of potential anomalies.

Anomaly detection is widely used in smart cities, especially in intelligent transportation systems. The intelligent transportation system discussed in this paper is an artificial intelligence-based technology that aims to detect intelligent traffic anomalies. By learning the relationships between sensors, we could detect anomalies from sensors data^3–5^. However, traffic anomalies usually exhibit complex forms due to two aspects: high dimensionality, sparsity, abnormal scarcity (i.e., the need to correlate time and space, including speed or flow), and difficulty in capturing the hidden relationship between nodes (i.e., spatial modeling in the face of different data sources with varying degrees of anomalies in density or distribution and scale)^6,7^. Therefore, it is important to explore ways to capture complex inter-sensor relationships and detect anomalies from node relationships. Several methods are based on Generative Adversarial Networks (GANs) based method^3^. However, the generator of GANs may be ineffective in fully capturing the hidden distribution of the data, which leads to a high false alarm rate and miss alarm rate due to the combination of the Binary CrossEntropyLoss (BCE) loss function. Most previous methods for anomaly detection are variants of Long Short Term Memory (LSTM)^8,9^, such as FC-LSTM^10^, which focuses on capturing both the various static factors and dynamic interactions that affect traffic flow. Moreover, there exists a category of networks, such as TCNs (temporal convolutional networks), designed to address temporal dependencies, which can capture global temporal information^11^. However, TCNs may not be as flexible in the context of traffic timing due to variations in the amount of historical information needed for model predictions across different domains. When TCNs (temporal convolutional networks) face a dynamic transportation network, their performance may be poor because their perceptual field is not large enough to describe the dynamics, complexity, and capture the global contextual information^12^.

The most advanced approach employs a graphical convolutional neural network (GNN) for spatial modeling reuse and combines LSTM to deal with anomaly prediction in time series^3^. There is also a method of passing adversarial training, learning the spatiotemporal features of traffic dynamics and traffic anomalies, respectively^13^. Existing methods of anomaly detection using graph convolutional neural networks (GCNs) do not well-address data sparsity and capture unseen nodes and the spatiotemporal correlation between nodes in the traffic network.

To solve the above problem, we propose a mirror temporal convolutional module (MTCM) to capture the anomalous information related to input data and hidden dynamic nodes in traffic networks. We mainly design two modules in MTGAE: mirror temporal convolutional module (MTCM) and graph convolutional gate recurrent unit cell (GCGRU CELL). Combined with self-adaptive, the MTCM can efficiently input into its modules in the face of sections of varying lengths in the dataset. MTCM explores the potential association between nodes and nodes by learning the complex hidden relationships and dependencies between nodes in traffic networks. GCGRU CELL module fully uses the existing prior knowledge (historical data). It captures road information, hidden node relationships, and dependencies for anomaly information redistribution, thus allowing us to obtain anomaly information more easily. We summarize the contributions of this paper as follows:

- We propose an anomalous detection framework called MTGAE, which maximizes the exploration of possible anomalies between nodes in the complex interdependencies, and better captures the hidden features between node-to-node in the traffic network.
- We construct a mirror temporal convolutional module, which is self-adapt to dataset and captures and cascades the hidden information between nodes by maximizing the breakthrough of the perceptual field of view of TCN.
- We propose the GCGRU CELL module, which captures long-term and short-term dependent anomalies in the traffic network space-time and maximizes the extraction of spatiotemporal features and possible anomaly information by cooperating with MTCM.

## Related work

In this section, we introduce the graph convolution networks, temporal convolutional networks, and autoencoder-based anomaly detection.

### Graph convolution networks

Recently, Graph Neural Network (GNN) variants, such as Graph Convolutional Networks (GCN), have demonstrated ground-breaking performances on many deep-learning tasks. In addition, it is modular, scalable, stronger in generalization ability, and explores insights that direct further research^14^. GCN captures the complex dependencies of node embeddings through information across vertices^15^. Due to these powerful features, in variants of GCN, the sensors on the road of the traffic network are considered nodes in intelligent transportation, and each node’s traffic speed or flow rate is regarded as a dynamic input feature. Among them, the Graph attention network (GAT) updates the node features through a pairwise function between the nodes with learnable weights^16^. However, it only computes one restricted form of static attention. To address this limitation, GATv2^17^ introduces dynamic attention alongside static attention, allowing for more dynamic and adaptive computation of graph attention. In the subsequent development of the GCN, CorrSTN^18^ effectively incorporates correlation information into the spatial structure. PDFormer^19^ captures both short-range and long-range spatial dependencies by utilizing various graph masking, which enables the learning of dynamic urban traffic patterns and overcomes the restriction of modeling spatial dependencies statically. Moreover, STAEformer^20^ takes into account the intrinsic spatial-temporal relationships and temporal ordering information in traffic time series. These methods are widely used in traffic forecasting, while graph embedding for traffic anomaly detection is less studied. For example, ST-Decompn solves the legal problem caused by changes in location and time in traffic cities through decomposition, as well as anomalies that may show up differently in the face of different datasets^21^, ConGAE detects traffic anomalies using semi-supervised frameworks such as autoencoders only for OD (origin-destination pairs) datasets on data washing and high dimensionality^13^. Besides, Graph Convolutional Adversarial Network (STGAN) uses adversarial training, which is divided into three modules to capture different features respectively: the recent module for local, the trend module for Long-term, and the external module for other traffic dynamics and anomalies, but the unsupervised learning, like an adversarial neural network, brings instability for anomaly detection^3^. Influenced state of the art, we borrowed the graph convolutional gated recurrent unit (GCGRU)^22^ simultaneously to solve the problem of Spatiotemporal characteristics of traffic anomalies. Our work is focused on the traffic anomaly prediction capabilities of GCN.

Graph autoencoders (GAEs) are a kind of unsupervised learning method, which means they map nodes to a potential vector space through an encoding process, reconstructing graph information from the vector to generate a graph similar to the original one (decoding)^15,23^. For example, ADN^24^ is a graph autoencoder structure and achieves information diffusion through alternating spatial and temporal self-attention. Due to the power of GAE^25^, it is widely used in different research directions, such as link prediction^26–30^, graph clustering^31,32^, hyperspectral anomaly detection^33^. While the traditional GCN takes node features and adjacency matrix as input and node embedding as output, GAEs compresses the node embeddings of all nodes in a graph to a single graph embedding to obtain information about the context.

#### Temporal convolutional networks

Earlier research methods focus on traffic-related problems but have shown significant inaccuracies in anomaly prediction. Deep learning has gradually dominated time series prediction tasks with sophisticated data modeling capabilities and autonomous learning abilities in recent years. Most studies in the field of transportation rely on gated linear Unit (GLU)^34^, or gated recursive units (GRU)^35^ to capture the dynamic temporal correlation of time series data. Moreover, based on the transformer architecture, STGM^36^ introduces a novel attention mechanism to capture both long-term and short-term temporal dependencies. Temporal convolutional networks (TCNs) also have significant advantages in addressing temporal dependencies, especially in time series prediction tasks. However, most traffic flow anomaly prediction frameworks use the original Temporal Convolutional Network (TCN)^37,38^ structure without modification, and traffic anomaly detection is still under-explored. In this study, we have enhanced the TCN to better detect anomalies within this domain, allowing for a more comprehensive analysis of time series data.

#### Autoencoder-based anomaly detection

The autoencoder, an unsupervised neural network, has seen significant success across various fields. This success is largely due to its superior ability to discriminate between abnormal and regular inputs, making it widely used in anomaly detection^39–44^. In the field of graph convolutional networks (GCN), GCN-based autoencoders are also employed for anomaly detection^45–48^. They are mainly studied in graph embedding, which is consistent with the direction of our work, thanks to the network structure of the graph, which can connect various points in the intricate world for anomaly detection.

## Methodology

Although many traffic anomaly detection methods have achieved optimal performance, they often overlook the hidden relationships between nodes during the detection process. For instance, traffic congestion during peak periods upstream can impact downstream traffic. This oversight results in many models lacking the ability to capture long-term temporal correlations, spatial characteristics, and high periodic trends. To address this, we aim to identify abnormal information and potential anomalies in the complex interdependencies among nodes in traffic networks. Consequently, we propose a traffic anomaly detection framework, MTGAE, with node interaction (see Fig. 2).

MTGAE consists of two main modules: MTCM and GCGRU CELL. The original input first passed through an adaptive process. This allows our module to better self-adapt to existing datasets by converting graph signals in low-dimensional spaces into potential vectors in high-dimensional spaces. Then we construct MTCM and GCGRU CELL. Specifically, we built MTCM to expand the hidden information in spacetime. MTCM internally expands *x* to the latent variables $x_m$ by mirror flip, and increases dilation factors and generates the hidden states *H* to capture both long-term spatiotemporal complex dependencies combining with TCN. Meanwhile, we built GCGRU CELL module to capture long-term and short-term 84 dependent anomalies in the traffic network. It combines original inputs and the hidden spatiotemporal states *H* as prior information. We first redistribute it through the Gaussian kernel module but without changing the overall structure of the traffic network (see in Fig. 2), then combine with our GCN modules to extract more spatiotemporal information. Subsequently, based on the output of the first GCGRU CELL, the spatio-temporal information $h_1^{(t)}$ and MTCM’s hidden information *H*, the second GCGRU CELL module adds more hidden details to correct the defects generated. Finally, we link the reconstructed results with the loss function to determine whether there are anomalies. In this section, we introduce the details of the MTGAE.

### Problem definition

**Figure 1:**
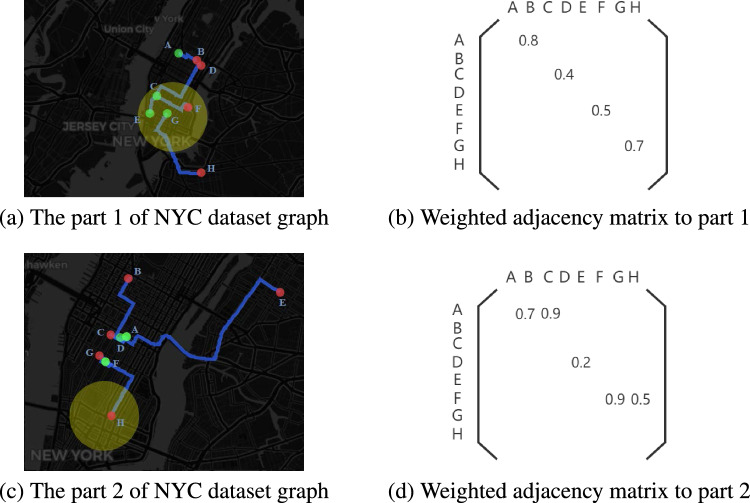
Illustration of two parts of NYC dataset graph (first column) and corresponding weighted adjacency matrix (second column) corresponding to scaled inverse travel times between points on the graph. Note that the mobility data is modeled as a series of time-dependent directed weighted graphs. (**a**) A portion of the visualizable NYC mobility data. (**b**) the adjacency matrix corresponding to subfigure (**a**). (**c**) Another portion of the visualizable NYC mobility data, but it is not part of subgraph (**a**). (**d**) the adjacency matrix corresponding to subfigure (**c**).

In this paper, traffic anomaly is monitored and detected in discrete time series $T \in (t_1,t_2,\ldots ,t_n)$. We denote the adjacency matrix representation graph as $G(T) = (V, E, W)$ where *V* indicates different nodes, such as two nodes $v_i$ and $v_j$, *E* denotes the set of edges between two nodes and *W* is the weighted adjacency matrix. A larger weight between two nodes means they are closer in the road networks and vice versa (see Fig. 1). Given $G(T) = (V, E, W)$, we aim to find the abnormal event $t_a \in T$ in the graph *G* that disrupts the regular traffic operation.

We aim to find the event $t_a \in T$ in the graph *G* that disrupts the regular traffic operation. We get the hidden state through a specially designed contextual encoder, embed the information as a coded low-dimensional embedding, and then decode it to derive the average reconstruction error that minimizes the weighted adjacency matrix. It should be noted that our model is specifically trained using data representing normal traffic conditions. Consequently, when an anomaly occurs in the traffic operation, it deviates significantly from this ’normal’ baseline. This deviation is captured as a high reconstruction error by our model, effectively indicating the presence of an anomaly.

### Encoder

**Figure 2:**
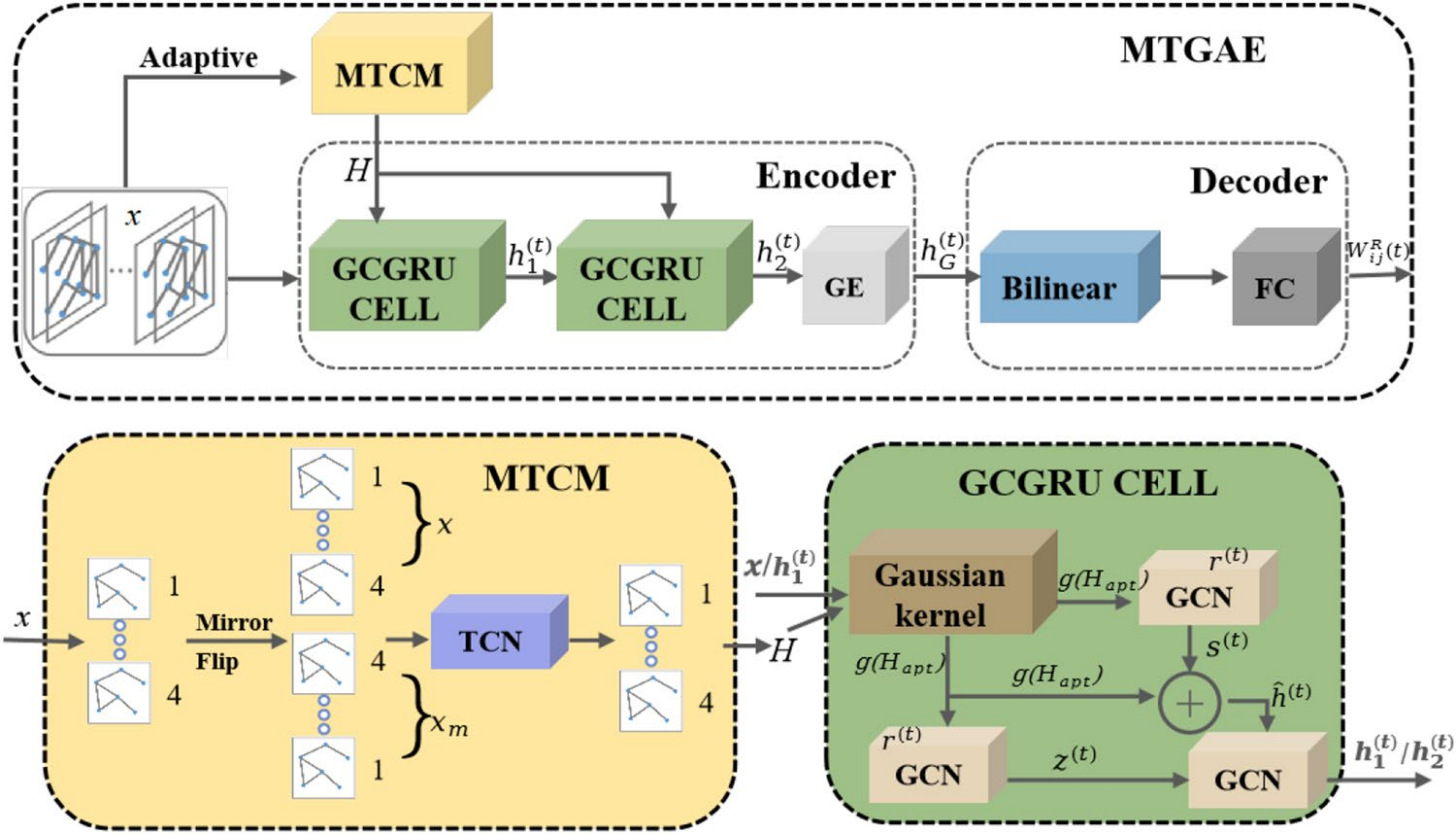
The architecture of the MTGAE. The architecture consists of two main components: an Encoder and a Decoder. The Encoder includes an adaptive process, the MTCM, and the GCGRU CELL. The MTCM is designed to effectively capture relevant information from data of variable length. It incorporates the TCN for processing data that has undergone a ’mirroring’ transformation, adjusting the length of mirrored data back to its original state prior to input. The GCGRU CELL, comprising a Gaussian Kernel and Graph Convolutional Networks (GCNs), is instrumental in mapping finite-dimensional data to a higher dimensional space. This mapping aids in anomaly detection while preserving data distribution. The GCNs within the GCGRU CELL leverage the GRU architecture to extract spatial-temporal dependencies. Lastly, the Decoder’s primary function, facilitated by the Bilinear module, is to resample features derived from the Encoder’s output, enhancing the overall data interpretation process.

Our encoder process comprises three steps: the adaptive process, the mirror temporal convolutional module (MTCM), and the graph convolutional neural network recurrent cell (GCGRU CELL). Initially, the original data, denoted as x, passes through the adaptive process, and MTCM is constructed to capture the evolving states that are not visible in the spacetime continuum among the road network nodes over time. In the GCGRU CELL, based on prior knowledge of the hidden states H from MTCM, our GCN layer, through the Gaussian kernel module, explores potential anomalies in the complex interdependencies between nodes. The encoder is trained to learn up to 24 hours in a day and 7 days in a week, facilitating interaction between the GCGRU CELL and a full connection (refer to Fig. 2). Finally, the graph embedding $h_2^{(t)}$ is applied.

#### Mirror temporal convolutional module (MTCM)

**Figure 3:**
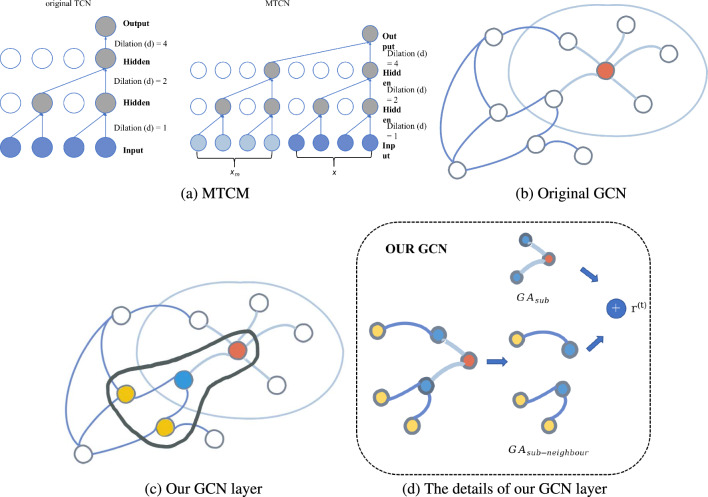
(**a**) The illustration explains: MTCM uses a hole convolution kernel with a size of 2. The left $x_m$ is the mirror image feature of $x$, uses the expansion factor K, selects the input of each k step, and then uses 1D convolution. (**b**) The figure explains how an embedded node and surrounding embedded nodes are connected through GCN, where the orange node is the original node, and its neighbour nodes are white and enclosed in the ellipse. (**c**) The figure explains ur GCN layer is different from the original GCN, our GCN layer can associate more sub-nodes. (**d**) The diagram shows how our GCN layer is associated with its child nodes (blue nodes) through the example orange node, and then the child nodes (blue nodes) spread to its child nodes (yellow nodes).

Inspired by TCN^11^ (see Fig. 3a), we proposed a superior module named MTCM that wides application in traffic prediction. Although TCNs can use the extended convolution to expand the perceptual field, they are weaker than advanced networks (e.g., Transformer) which can use correlation information of arbitrary length. Moreover, TCNs need strong adaptability to different historical information, which may have uneven predictive power and perceptual field. To overcome the above situations, we adapt the TCN before transmitting the traffic network features to reduce the fluctuation of different historical information on the ability of the TCN. We then perform a mirror flip to further preserve the features and capture the complex hidden relationships and dependencies between nodes in the traffic network. This explores the potential associations between nodes. Furthermore, thanks to the one-dimensional convolution of the TCN, we can keep the output sequence consistent with the original input in length. Finally, this output sequence will be passed as the subsequent hidden state *H*. More formally, for a 1-D sequence input $\varvec{x} =\{x_1, x_2, x_3,\ldots x_i\} \in \mathbb {R}^i$ and a filter $\varvec{f}: \{0, \ldots , j - 1\} \rightarrow \mathbb {R}$, k is the kernel size (the kernel size in the Fig. 3 is 2), and *d* is the causal factor (see Fig. 3a). The dilated convolution operation *f* on element *x* of the sequence is defined as:

1 $$\begin{aligned} (x_{m}\oplus x)*f(j)=\sum _{i=0}^{k-1}f(i)\cdot j-d \cdot i \end{aligned}$$

where $x_m$ is the sequence input in mirror flipping, $\oplus$ denotes concatenate. This further increases the range of perceptual field and prevents more historical data from being lost in the process of inflated convolution.

#### GCGRU CELL

**Figure 4:**
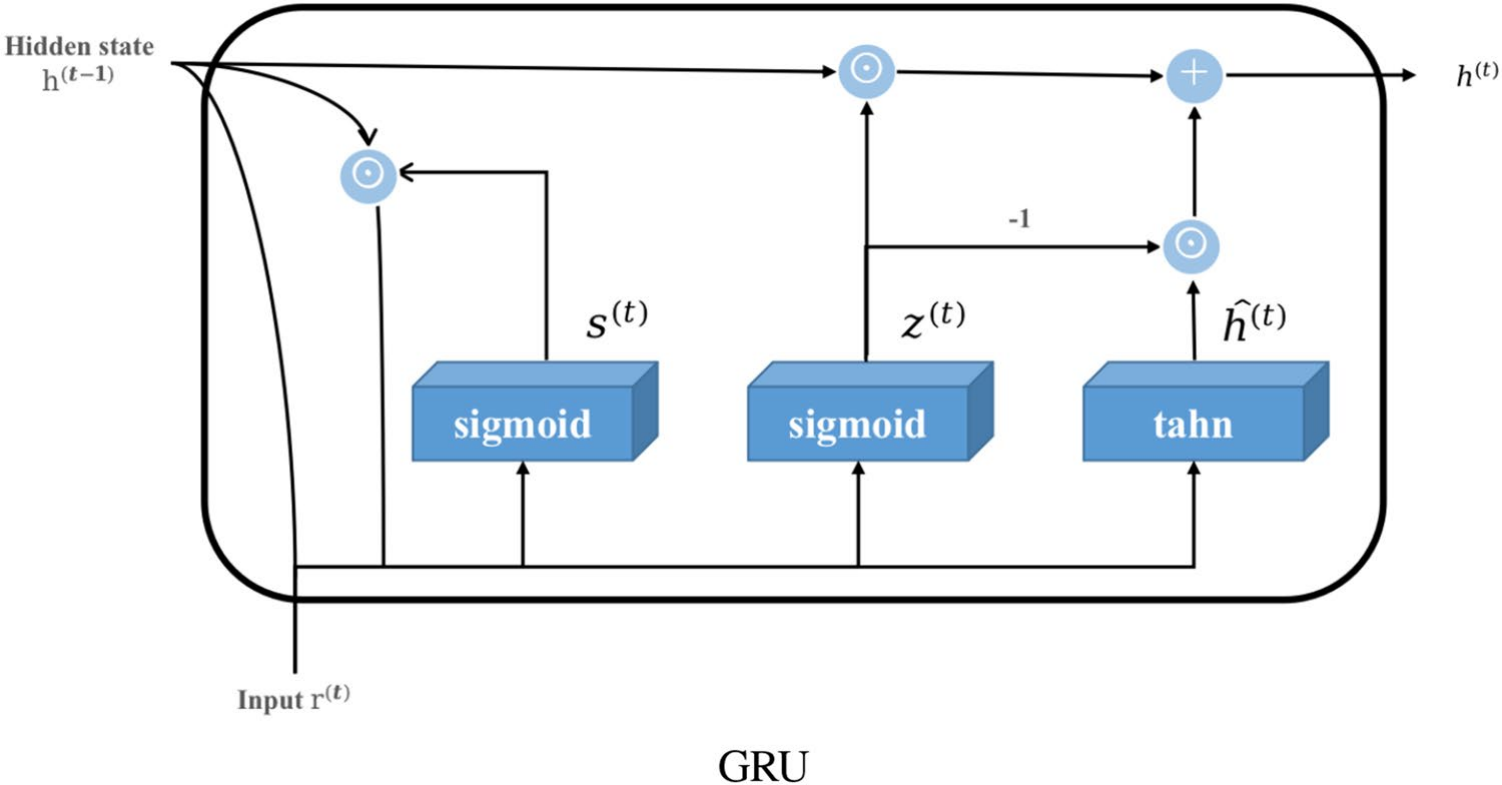
The image shows the GRU architecture, on which we were inspired to change the gating unit of GRU to GCN, giving it the same ability as GRU to capture short-term and long-term dependencies in a sequence. The $s^{(t)}$ update gating and $z^{(t)}$ reset gating are reflected in the derivation equations of GCGRU in this paper.

It mainly includes the Gaussian kernel module and GCN layer. We did not adopt GRU model (as shown in Fig. 4) but construct the GCN model inspired by GRU after the Gaussian kernel module. In GCGRU CELL, we replace the original gated cyclic unit of GRU to our GCN, which has the following two significant: the reset gate helps to capture short-term dependencies in the sequence, and the update gate helps to capture long-term dependencies in the sequence. This effectively predicts both long-term and short-term traffic network cycles, and combine with Gaussian kernel processing and prior knowledge *H* (hidden information of MTCM), GCN can capture anomaly information and possible anomalies in complex interdependencies among nodes while predicting. Unlike image data, Graph convolution is an essential operation to extract a node’s features. Figure 3b gives examples of an origin node (orange node) to take the average value of the node features within its neighbours (white nodes in ellipse).

**Figure 5:**
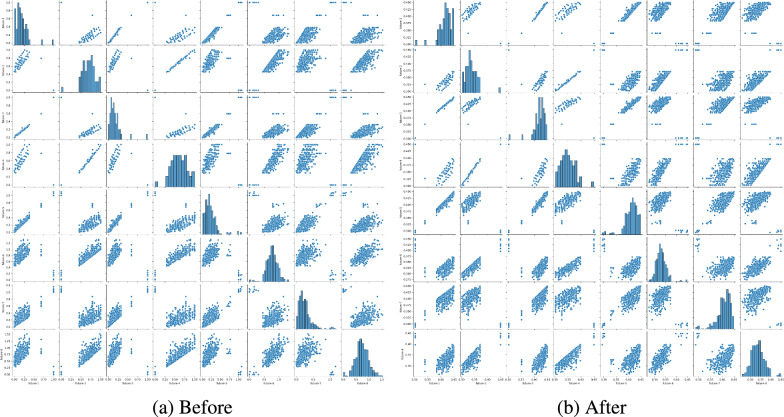
We extracted the intermediate variables before and after using the Gaussian kernel module to visually demonstrate this module’s importance in our model. (**a**) Before the Gaussian kernel module. (**b**) After the Gaussian kernel module.

**(1) Gaussian kernel module** To further enhance the anomaly detection capability of our module, we employ Gaussian kernel function. It could maintain the ability of high-dimensional data distribution characteristics, which is crucial for traffic network anomaly detection. Specifically, Gaussian kernels facilitate the mapping of data from its original space to a higher-dimensional feature space where complex traffic network patterns and potential anomalies are more easily identified and processed. Moreover, Gaussian kernel exhibit the stability: It could manage minor fluctuations by adjusting learned scale parameter $\sigma$ (see Eq. 2) or utilizing a minimax strategy^49^, thereby ensuring more stable anomaly detection results. In summary, embedding Gaussian kernels in the GCGRU CELL module aims to enhance the model’s performance and accuracy in detecting anomalies within complex traffic networks. Experimental data demonstrate that using Gaussian kernels to alter the data distribution effectively improves the accuracy of traffic anomaly detection (see Table 3). Building on this foundation, we further explored the anomaly detection capabilities of the Gaussian kernel module. As depicted in Fig. 5, we performed an intermediate variable exploration of the eight feature points generated by 490 edges entering the GCGRU CELL. This demonstrates the stability and the data mapping capability of our module by conducting visualization operations on intermediate variables before and after integrating the Gaussian kernel module into the GCGRU cell. Throughout the experimentation, the overall structure of the data remains unchanged, ensuring consistency and reliability. Our GCGRU CELL receives two input modes. The first input is from the original input *x* after adaptation and receives the hidden information *H* from the MTCM. Then set the input as $\varvec{H_{apt}}=x+H$. The second input is the output of the first GCGRU CELL $h_ 1 ^ {(t)}$, which also receives hidden information *H*. We also set this input as $\varvec{H_{apt}}=h_ 1^{(t)} + H$. Then, the formula calculated by the Gaussian kernel module is as follows:

2 $$\begin{aligned} {\textrm{g}}(H_{apt}) = \frac{1}{{\sigma _i \sqrt{2\pi } }}{e^{ - \frac{1}{2}{{\left( {\frac{{H_{apt} - i }}{\sigma _i }} \right) }^2}}}, \end{aligned}$$

where $g(H_{apt})$ is generated based on the learned scale $\sigma$ (we usually set the value between 0.5 and 1) and the *i*-th element $\sigma _i$ corresponds to the *i*-th time point. Specifically, for the *i*-th time point, its association weight to the $H_{apt}$-th point is calculated by the Gaussian kernel.

**(2) GCN layer** Generally, the traffic network is presented as a weighted digraph. Traditional graph convolution networks only operate on adjacent nodes, which results in better short-term prediction than long-term prediction. Therefore, the spectral graph theory is used in this paper. Let $\varvec{G}=(V, E, W)$ and establish spectral matrix $\varvec{L}=I - \hat{D} ^ {- 1/2} \hat{A} \hat{D} ^ {- 1/2}$, where *I* is the identity matrix and $\hat{D}$ is the degree matrix, $\hat{A}$ is the adjacent matrix. To explore deeper and more complex traffic networks, we extend the graph convolution network to a higher level and divide the traffic graph *g*(*x*) sent by the Gaussian kernel module into subgraph $GA_ {sub}= \{g (H_{apt1}), g (H_{apt2})\ldots g (H_{aptn}) \}$, and the subgraph considers its neighbour nodes $GA_{sub-neighbour}$, which achieves more high-order information aggregation.

3 $$\begin{aligned} r^{(t)} = ReLU(GA_{sub}^{(t-1)} \cdot {W_1^{(t)}} + GA_{sub-neighbour}^{(t-1)} \cdot {W_2^{(t)}}), \end{aligned}$$

where $W_i^{(t)}$ represents learnable weights, and $r^{(t)}$ denotes the computed results of graph convolution as time *t*increases.

In a separate aspect, the use of GRU^49^ simplifies the model, reducing complexity and enabling a faster, more effective characterization of sentence semantics. Compared to LSTM, GRU reduces the number of gating parameters, utilizes fewer training parameters, requires less memory, and offers faster execution and training. Owing to these advantages, our model adopts the GRU architecture over the traditional LSTM approach. We have transformed the gating unit into a graph convolution layer, as outlined in Eq. (3). This adaptation allows the GRU architecture to imitate the gating unit effectively. Consequently, the GCN layer can discern more hidden states from data processed by the Gaussian kernel module, capturing the dynamic spatial correlations within the traffic network and identifying previously unseen network connections. Formally,

4 $$\begin{aligned} \begin{aligned} s^{(t)}&= \sigma ({W_z}{r^{(t)}} + {U_z}{g(H_{apt})}), \\ z^{(t)}&= \sigma ({W_s}{r^{(t)}} + {U_s}{g(H_{apt})}), \\ {\widehat{h}^{(t)}}&= \tanh {({W_{{r^{(t)}}}} + U({s^{(t)}} \odot {g(H_{apt})}))}, \\ {h^{(t)}}&= {z^{(t)}} \odot {h^{(t - 1)}} + (1 - {z^{(t)}}) \odot {\widehat{h}^{(t)}}, \end{aligned} \end{aligned}$$

where $h^{t-1}$ is the previous memory state, $W_z$, $W_s$ and $U_z$, $U_s$, *U* are the weight parameters, $r^{(t)}$ is the current feature input, and $\sigma$ is a sigmoid activation function. We combine GCN and GRU to capture the long-term dependencies between nodes in the graph.

**(3) Graph Embedding (GE)** We construct a time embedding (referred to as the GE module in Fig. 2) after the second GCGRU CELL to effectively capture the intricate weekly and hourly periodicity inherent in the mobility data. The time embedding consists of two components: $h_{hour} \in R^{day}$ represents the time of day embedding, and $h_{day} \in R^{week}$ represents the day of week embedding. For example, at a specific time t (e.g., 13:00 on Saturday, July 30), we use $h_{hour}$ (i.e., 13:00) and $h_{day}$ (i.e., Saturday) as the time embeddings. These embeddings serve the purpose of incorporating additional temporal information as context for the conditioned encoder and decoder. By incorporating these temporal factors as graph embeddings ${h_G}^{(t)}$, the model could accurately capture and represent the patterns and variations in mobility data associated with different times and days.

5 $$\begin{aligned} \begin{aligned} {\widetilde{h}_G}^{(t)}&= \oplus (h_1^{(t)},h_2^{(t)},\ldots ,h_n^{(t)},{h_{hour}}(t),{h_{day}}(t)),\\ {\widetilde{u}_G}^{(t)}&= {{\hat{U}}^{({\textrm{t}})}}({{\hat{U}}^{({\textrm{t}})}}({\widetilde{h}_G}^{(t)})), \\ {h_G}^{(t)}&= {\mathop {\textrm{Re}}\nolimits } LU({U_G}{\widetilde{u}_G}^{(t)}), \end{aligned} \end{aligned}$$

where ${h_G}^{(t)}$ is the graph embedding at time *t*, $\hat{U}$ from the formula 4 and $U_G$ is weight matrix.

### Decoder

In the decoder, we begin with information extraction about the node embedding from the graph embedding ${h_G}^{(t)}$. For each pair of node embeddings $(v_i, v_j)$, we embed the time information ${h_{hour}}^{(t)},{h_{day}}^{(t)}$ into the information of each pair of nodes and compute the corresponding weight $w_{ij}$ in the weighted adjacency matrix. We then combine these node embeddings and time embeddings to form a graph embedding information ${\widetilde{h}_G^{\prime }}{(t)}$ that varies over time *t*. It contains both the information of the nodes and the time information (that is, the embedding includes the collective features of all nodes in the graph at that moment *t*). Subsequently, a fully connected layer is used to process this graph embedding, to recover useful vector representations from it. After processing by the fully connected layer, the vectors *i* and *j*, corresponding to ${h_i}^{(t)}$ and ${h_j}^{(t)}$, are unstacked to recover the embedding of each individual node at time *t*. Consequently, the outcome of this process is the embedding representation ${h_n}^{(t)}$ of a particular node *n* under specific time *t* conditions. Finally, to obtain the reconstructed edge weights, we first used the *ReLU* activation function to process the graph embeddings, resulting in a feature vector ${\hat{W_{ij}}(t)}$ that has undergone a nonlinear transformation. Then, the reconstructed edge weights ${W_{ij}^{R}(t)}$ are obtained from the feature vector ${\hat{W_{ij}}(t)}$ and the *Sigmoid* function.

The presence of a bilinear module in the decoder is significant. The bilinear module applies a transformation to the incoming data, serving two main benefits: 1) The bilinear module ensures that edge weight predictions consider directionality. In the directed graph, the edge weight from node *i* to node *j* could differ from the weight from node j to node i. 2) The bilinear module employs the formula $w_ij={h_i}^{(t)}A{h_j}^{(t)}$ to calculate the edge weights, where *A* is a learned parameter. This approach enables the model to distinguish edge weights based on direction, more accurately depicting directed graph relationships.

6 $$\begin{aligned} \begin{aligned} {\widetilde{h}_G^{\prime }}{(t)}&= ReLU({{dec}_G}Concat({h_G}^{(t)},{h_{hour}}^{(t)},{h_{day}}^{(t)})),\\ \{ {h_1}^{(t)},h_2^{(t)},\ldots ,h_n^{(t)}\}&= unstack({\widetilde{h}_G^{\prime }}{(t)}),\\ {\hat{W_{ij}}(t)}&= ReLU({dec_1} Concat({h_i}^{(t)}, {h_j}^{(t)})), \\ {W_{ij}^{R}(t)}&= Sigmoid({dec_2} {\hat{W_{ij}}(t)}) \end{aligned} \end{aligned}$$

where $dec_G$ is weight matrix, $dec_1$ is the weight matrix of feature vector ${\hat{W_{ij}}(t)}$ and $dec_2$ is the weight matrix of ${W_{ij}^{R}(t)}$. The Sigmoid ensure the output ${W_{ij}^{R}(t)} \in [0, 1]$.

### Loss function

We use the mean squared error (MSE) as the loss function, a measure of the difference between the actual value *y* and the predict $\hat{y}$, to evaluate our model. Formally:

7 $$\begin{aligned} \begin{aligned} {Loss({W_{ij}^{R}(t)}, W_{ij}) = \frac{1}{n} \sum ^{n}_{i=1} ({W_{ij}^{R}(t)}-W_{ij})^2}, \end{aligned} \end{aligned}$$

where *i* is the value of each point in the sequence. And the reconstructed weights are $W_{ij}^{R}(t)$ and the actual weights are $W_{ij}$. During testing, the loss function Eq. (7) for each testing instance is used as its anomaly score.

## Experiments

### Datasets and implementation

To ensure the model’s credibility, we focused on general datasets that target traffic anomaly detection in our experiments. We verify our MTGAE method on two public traffic network datasets.

- **PEMS-BAY dataset:** It is collected in real-time from nearly 40,000 individual detectors spanning the freeway system across all major metropolitan areas of California^50^. The dataset comprises 365 sensors located in the bay area, and it contains traffic data recorded from April to May 2014. For our analysis, we selected a subgraph of six sensors, each with recorded speed and traffic flow information pertaining to our network. Furthermore, we extended the duration of each traffic incident from CHP (CHP Traffic Incident Information https://www.chp.ca.gov/traffic), by one hour to account for the impact of traffic accidents.
- **New York City (NYC) taxi dataset:** The New York City (NYC) taxi trips dataset is publicly released by the Taxi and Limousine Commission (TLC). We use it to record the time and location of each taxi pick-up and drop-off and pool the records formed for each hour of that taxi into a matrix. This dataset includes six months of data, from January 2019 to March 2019. Since the NYC dataset lacks exception tagging points, we utilized exception injection to add exceptions into the timing of the dataset^51,52^.

### Baselines

To validate our method’s effectiveness in anomaly detection within the NYC dataset. We obtained these methods from their official public code repositories and employed their optimal experimental setups, running all models on the NYC dataset to guarantee fairness:

- **Con-GAE**^13^: The method was developed to tackle the challenges posed by extreme data sparsity and high dimensionality, specifically to address anomalies in traffic conditions. Moreover, It utilizes context-enhanced graph autoencoders to enhance the effectiveness of anomaly detection.
- **SuperGAT**^53^: A self-supervised graph attention network, uses edge information to guide attention learning. SuperGAT analyzes two common attention forms, revealing their limitations in capturing label agreement and edge presence, and proposes enhanced attention mechanisms tailored to graph characteristics.
- **EG**^54^: The Efficient Graph Convolution (EGC) method is an isotropic Graph Neural Network (GNN) architecture.EGC outperforms comparable anisotropic models like GAT and PNA in terms of accuracy and efficiency. This finding challenges the prevalent belief that anisotropic GNNs are inherently superior.
- **GraphGPS**^55^: A modular and scalable framework designed to build graph transformers, integrating message passing with global attention. This framework also categorizes positional and structural encodings, thereby injecting useful inductive biases. GraphGPS demonstrates state-of-the-art performance in various graph learning tasks and scales effortlessly to thousands of nodes.
- **GATv2**^17^: Graph Attention Networks (GATs) are limited by their computation of restricted “static” attention, inhibiting their ability to dynamically prioritize neighbors. To overcome this limitation, GATv2 alters the order of operations in the scoring function, enabling more expressive dynamic attention.
- **Dir-GNN**^56^: The method enhances message passing neural networks (MPNNs) by incorporating edge directionality and conducting distinct aggregations for incoming and outgoing edges. Moreover, It significantly betters learning on heterophilic graphs, where neighboring nodes often have different labels, and maintains performance on homophilic graphs, characterized by label-sharing neighbors.
- **PMLP**^57^: The method introduces propagational MLPs, which employ MLP architecture for training and add message passing layers before inference. This approach bridges the gap between MLPs and GNNs, achieving performance that is comparable to or surpasses that of GNNs. It demonstrates the effectiveness of GNN architectures for generalization, even without training in a graph context. Additionally, PMLPs offer faster and more robust training than GNNs.

### Experimental setups

Our experiments were conducted using a GPU 2080TI and an Intel(R) Core(TM) i7-7700K CPU @ 4.20GHz. Considering the anomaly problem, we experimentally used anomaly injection, randomly selecting time slices $\theta$ in each sequence to inject anomalies. Then extract a portion of the time series corresponding uniformly distributed time slices $\theta$ for perturbation factors for anomaly perturbation (e.g., 10 am for 10 pm). In this experiment, we set three pollution ratios and magnitudes $\alpha$, $\beta$, and $\gamma$ on the data set NYC. Anomalies in traffic networks are mainly divided into two types^58,59^: (1) Spatial anomalies: where the current traffic conditions are inconsistent with normal traffic conditions (for example, the flow of traffic vehicles is inconsistent with the normal flow of travel in the past). (2) Temporal anomalies: where the current traffic conditions conform to the normal spatial pattern, but not to the current time. In this paper, we perform some anomaly handling on the dataset: Let $\gamma$ represent the proportion of time slices randomly selected for contamination, which is applicable to the injection of both spatial and temporal anomalies; Let $\alpha$ denote the proportion of origin-destination pairs selected for contamination; Let $\beta$ defines the range of the uniform distribution used to perturb the travel time. In fact, $\beta$ defines the magnitude of spatial anomalies, i.e., the maximum possible value of travel time perturbation.

In the experiments, we adjust the levels of pollution ratios and magnitudes ($\alpha$, $\beta$, and $\gamma$), to evaluate the effectiveness of anomaly detection under different scenarios. The specific steps are as follows: For spatial anomalies, we first randomly select a certain proportion ($\gamma$) of time slices and randomly choose a certain proportion ($\alpha$) of origin-destination pairs in each contaminated time slice, and then perturb the travel time of these pairs by factors drawn from the uniform distribution *U*($-\beta$, $\beta$). Temporal anomalies are created by randomly selecting a certain proportion ($\gamma$) of time slices and shifting the time in the data by 12 hours (e.g., changing 8 PM to 8 AM, and vice versa). We set $\alpha \in \{25\%, 50\%\}$, $\beta \in \{5\%, 10\%, 20\%\}$, and $\gamma \in \{5\%, 10\%, 20\%\}$.

For the training process, we initially set the epoch number at 150 and the batch size at 10 per epoch. In the previously mentioned day of the week and hour of the day metrics, set both $h_{day}$ and $h_{hour}$ to 100, and the dimension of the graph embedding we set to 150 and 50, respectively, the discard rate was set to 0.2, the learning rate is 0.001 by default. Then, we set the learning rate decay in the process, each time, the growth is 0.1 times the last learning rate so that the model can learn the parameters better. Finally, we selected the NYC datasets from January 8 to March 31, 2019, as the training set and extracted 10% from it for validation. We used the NYC datasets from January 1 to January 7, 2019, and a portion of the Uber Movement as the test set. Note that the sampling process was based on uniform distribution random sampling, and both training set and test set were mutually exclusive (i.e., the same data point would not appear in both the training set and test set).

In addition, ablation experiments were performed on the PEMS dataset to verify the effectiveness of the proposed module, which was evaluated using MAE metrics. The loss functions that MAE and RMSE are more credible test methods in some anomaly detection, especially in the traffic area^3^. Six epochs were set for training. Each period was divided into 128 batches, the generator loss function was 500, the learning rate was 0.001, and the decayed by a factor of 0.1 per epoch. In this dataset, we set the number of layers of TCN to 9 and transformed the head nodes in GCN to GAT to improve the model’s parallelism. In learning, we set the hidden layer to 64.

### Result and analysis

**(1) Comparison with state-of-the-art work** Initially, we compared our MTGAE model with some baseline models using the AUC as evaluation metric. The calculation of AUC considers both the classification ability of the classifier for positive and negative cases, which can still make a reasonable evaluation of the classifier in the case of sample imbalance. We fixed $\alpha =50\%$ and $\beta =10\%$ in the pollution magnitude and used the anomaly rate to compare the model’s ability to detect anomalies. It can be seen from Table 1 that our MTGAE is significantly better than the other models. Our model outperforms others by about 0.1–0.4 at different anomaly rates.

**Table 1:** Given fixed $\alpha$ and $\beta$, the AUC scores of different models with the fraction $\gamma$ of the time slices chosen to be polluted.

anomaly rate $\gamma$	5%	10%	20%
HA (1981)	0.728	0.687	0.711
RTC (2020)	0.736	0.765	0.790
AE (2002)	0.813	0.812	0.806
EncDec-AD (2016)	0.584	0.582	0.582
REBM (2018)	0.844	0.859	0.833
DAGMM (2016)	0.550	0.546	0.507
GraphSAGE (2017)	0.842	0.840	0.860
GCN (2016)	0.708	0.717	0.744
Con-GAE (2021)	0.903	0.908	0.913
SuperGAT (2021)	0.901	0.908	0.910
EG (2021)	0.883	0.902	0.912
GraphGPS (2022)	0.894	0.906	0.911
GATv2 (2022)	0.887	0.899	0.902
Dir-GNN (2023)	0.882	0.899	0.911
PMLP-layer128 (2023)	0.960	0.965	0.968
MTGAE	1.000	1.000	1.000

After that, we fixed the time slice $\gamma$ of pollution to study the abnormal magnitude to change the pollution magnitude differently. As shown in Table 2, we controlled $\alpha$ as 25% and 50% respectively, and $\beta$ was controlled as the same pollution magnitude under $\alpha$. We can see that our models are higher than the baseline but in the higher $\beta$. For example, the AUC of most models with $\alpha =50\%$ and $\beta =20\%$ is above 0.9, and most of the baseline models with $\beta =5\%$ are not performing well. Instead, they all gradually increase in AUC capacity after $\beta$ increases, while our model has an excellent performance in all aspects, so it is a more competitive model.

**Table 2:** Given fixed fixed $\gamma$, the AUC scores for anomaly detection. Results are shown under different $\alpha$ and $\beta$.

Spatial anomaly rate $\alpha$	25%			50%		
Anomaly magnitude $\beta$	5%	10%	20%	5%	10%	20%
HA (1981)	0.405	0.533	0.804	0.455	0.687	0.934
AE (2002)	0.294	0.572	0.936	0.405	0.812	0.994
EncDec-AD (2016)	0.410	0.483	0.727	0.452	0.582	0.896
GCN (2016)	0.443	0.564	0.844	0.498	0.717	0.966
DAGMM (2016)	0.511	0.527	0.567	0.525	0.546	0.639
GraphSAGE (2017)	0.381	0.627	0.963	0.491	0.840	1.000
REBM (2018)	0.389	0.633	0.958	0.491	0.859	0.997
RTC (2020)	0.626	0.699	0.863	0.648	0.765	0.942
Con-GAE (2021)	0.946	0.755	0.985	0.610	0.908	1.000
SuperGAT (2021)	0.904	0.910	0.911	0.893	0.908	0.911
EG (2021)	0.897	0.904	0.906	0.888	0.902	0.906
GraphGPS (2022)	0.900	0.909	0.911	0.890	0.906	0.911
GATv2 (2022)	0.894	0.901	0.902	0.885	0.899	0.902
Dir-GNN (2023)	0.893	0.902	0.904	0.883	0.899	0.904
PMLP-layer1 (2023)	0.895	0.902	0.905	0.884	0.900	0.905
PMLP-layer2 (2023)	0.880	0.887	0.888	0.871	0.885	0.889
PMLP-layer64 (2023)	0.916	0.924	0.926	0.906	0.922	0.926
PMLP-layer128 (2023)	0.960	0.967	0.968	0.952	0.965	0.968
MTGAE	1.000	1.000	1.000	1.000	1.000	1.000

**(2) Ablation study** In the ablation study, we evaluated several MTGAE variants to evaluate the effects of different parts of our MTGAE (see Table 3). The variants include: (i) MTGAE-gan: We used the framework of GAN instead of an autoencoder. (ii) MTGAE-ot: We adopted the approach of using an autoencoder and only employed the original TCN instead of our proposed MTCM. (iii) MTGAE-mt: We removed TCN and incorporated our proposed MTCM module. (iv) MTGAE-lstm: We removed the GRU and replaced it with the LSTM. (v) MTGAE-grumt: We removed the LSTM and replaced it with the GCGRU CELL. (vi) MTGAE-Transformer: We removed the GCGRU CELL and replaced it with the Transformer. (vii) MTGAE-gb: We incorporated the Gaussian kernel module into MTCM and GCGRU CELL, placing it in the final data processing stage of the GCGRU CELL. (viii) MTGAE: Our complete model framework. The study indicates that the basic TCN variant underperforms unless combined with the Gaussian kernel function for processing, which illustrates the importance of MTCM and GCGRU CELL for anomaly detection. Notably, incorporating a mirror into TCN significantly improves its efficacy in enhancing GCGRU CELL performance, this demonstrates superior ability in capturing both long and short-term memory and temporal information in time series.

**Table 3:** Ablation study.

Model	MAE	RMSE	Note
MTGAE-gan	3.076	6.770	Replace our model architecture with GAN
MTGAE-ot	3.150	6.880	The temporal convolution module without mirror
MTGAE-mt	3.019	6.713	The temporal convolution module with mirror
MTGAE-lstm	3.224	6.940	Replace GRU with LSTM
MTGAE-grumt	3.052	6.751	Add the GCGRU-Cell module to the architecture
MTGAE-Transformer	17.020	32.507	Replace our model architecture with Transformer
MTGAE-gb	3.088	6.780	The Gaussian kernel module as a post-processing step
MTGAE	2.990	6.679	Our method

**Figure 6:**
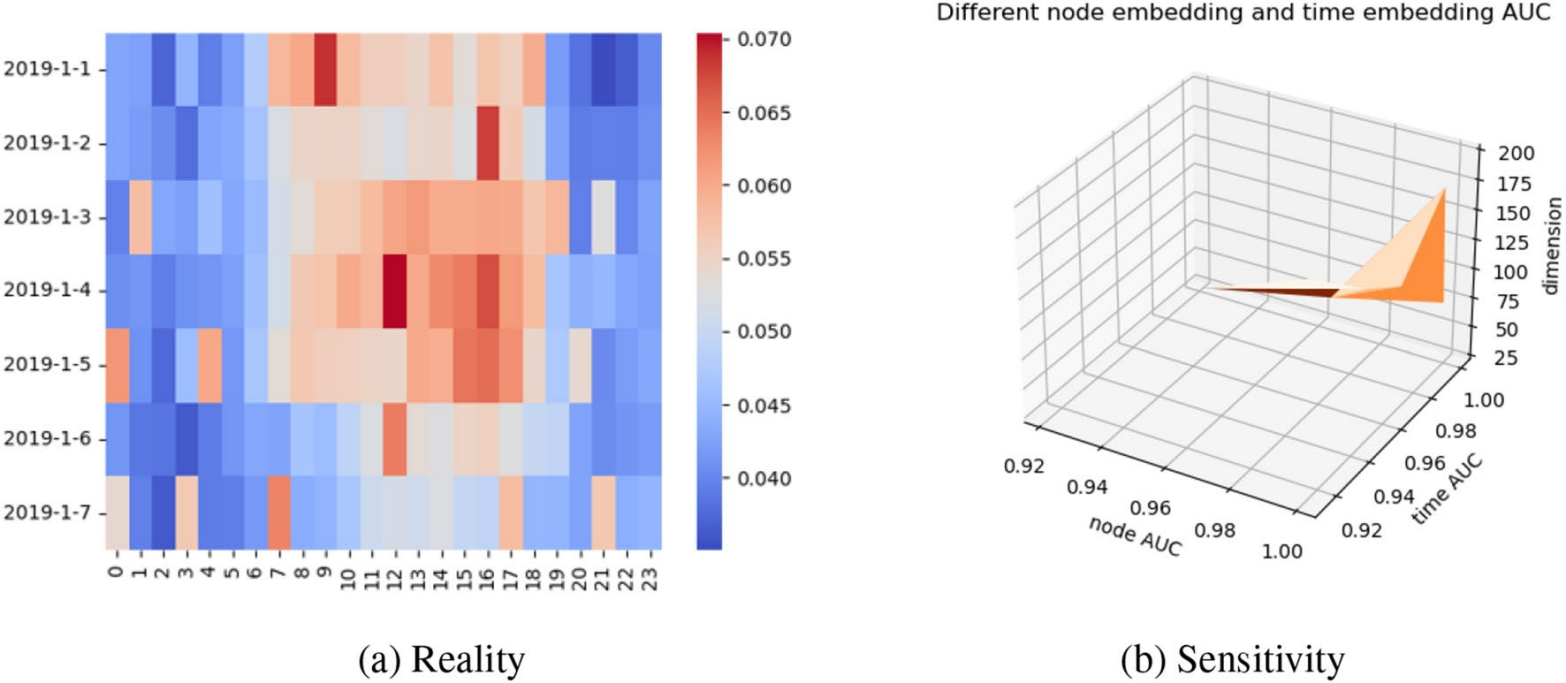
(**a**) Our model’s ability to detect traffic anomalies. The horizontal axis denotes an hour, the vertical axis denotes date, and the color depth indicates the possibility of traffic anomalies (reconstruction loss). (**b**) The sensitivity experiment of the model. It is guaranteed to be between 0.9 and 1.0 under different node embedding and time embedding.

**(3) Real world reflects abnormal traffic** We used the NYC dataset from January 1, 2019, to January 7, 2019, to test the real-world traffic situation to prove the effectiveness of our model. We used the reconstruction loss to represent the possibility of anomalies, as shown in Fig. 6. January 4 is Friday in the real world, and we can see that the possibility of anomalies in the afternoon distribution of this day is very intensive, from which we can infer that Black Friday Shopping is prone to traffic anomalies due to traffic jams.

**(4) Sensitivity analysis** To study how MTGAE varies for weekly, hourly, and node embedding, we put $\alpha =50\%$ and $\beta =10\%$ $\gamma =10\%$. We explored the model’s affectivity on spacetime, and we changed the dimension of node embedding to 25 to 200 (the dimensionality is acceptable for the first GCN and the second GCN) and the week and hour dimension of temporal embedding to 10 to 200 for training. As shown in Fig. 6b, our model does not change much, and the AUCs all remain between 0.9 and 1, indicating that our model works well in most environments. Moreover, we can further see that the AUC of our model is lower when the time node embedding is large than when the embedding is small.

**(5) Generalization ability** To explore the generalization ability of MTGAE, we performed experiments on a large-scale dynamic graph dataset DGraphFin in the financial domain^60^. It contains over 3.7 million nodes and 4.3 million dynamic edges. Nodes represent financial loan users, and directed edges represent emergency contact relationships. Each dimension represents 17 different elements of personal profiles, such as age and gender. Among the nodes in the dataset, 15,509 are categorized as fraudsters, 1,210,092 as normal users, and the remaining 66.8% of nodes (2,474,949 nodes) are registered users who have not borrowed from the platform. Based on the officially published baseline and code, we input the DGraphFin data into our MTGAE, then carry out feature learning through the 17 features in the structure of MTGAE, and finally divide into two categories (other baselines also divide into two categories) for anomaly detection, with results shown in the Table 4. Compered with the network specifically designed for DGraphFin dataset, experiments results illustrates that our MTGAE possesses certain generalization capabilities.

**Table 4:** The AUC scores of our model and other baselines on the DGraphFin dataset.

Model	AUC
GCN	0.707
MLP	0.719
GAT	0.733
GATv2	0.762
TGN	0.774
SAGE	0.776
MTGAE	0.768

## Conclusions

This paper proposes MTGAN, a spatio-temporal anomaly detection framework for traffic. In the encoder, we propose two modules: the Mirror TCN (MTCM) and a variant of GCGRU, namely the GCGRU CELL that captures correlations in spatial and temporal dimensions, and a practical approach: adaptive TCN. We then performed anomaly injection on the dataset by three contamination metrics and tested it on the NCY dataset. Experiment results show that our framework outperforms the baseline in traffic anomaly detection, particularly in aspects of sparsity and high dimensionality, thereby contributing to further research. In future work, we will explore additional extensions of MTGAE in more datasets and further explore methods for learning dynamic spatial correlations.

## Data Availability

The datasets analyzed during the current study are available in the GitHub repository, including the NYC dataset and the PEMS dataset, which can be found at https://github.com/yuehu9/Con-GAE and https://github.com/dleyan/STGAN, respectively. And the datasets used for testing the generalization capabilities: https://dgraph.xinye.com/dataset. Additionally, we collected and analyzed some of the data used in our experiments. The experimental data collected during the current study available from the corresponding author on reasonable request.
